# Transgene behavior in *Zea mays* L. crosses across different genetic backgrounds: Segregation patterns, *cry1Ab* transgene expression, insecticidal protein concentration and bioactivity against insect pests

**DOI:** 10.1371/journal.pone.0238523

**Published:** 2020-09-10

**Authors:** André Felipe Lohn, Miluse Trtikova, Ignacio Chapela, Johnnie Van den Berg, Hannalene du Plessis, Angelika Hilbeck

**Affiliations:** 1 Plant Ecological Genetics, Institute of Integrative Biology, Department of Environmental Systems Science, ETH Zürich, Zürich, Switzerland; 2 Department of Environmental Science, Policy and Management, University of California Berkeley, Berkeley, California, United States of America; 3 Unit for Environmental Sciences and Management, North-West University, Potchefstroom, South Africa; University of Texas at Dallas, UNITED STATES

## Abstract

Brazil and South Africa, countries with economies in transition, are characterized by a dual agrarian structure co-occurring, sometimes, alongside in the same region. Large-scale commercial farming produces crops for export to global markets on the one hand, and small-scale farming, on the other hand, provides for subsistence and local markets. In both systems, maize (*Zea mays*) is a key crop for these two countries. For the commercial system, maize is a commodity crop while for the small-scale system it is the prime staple crop. In commercial systems, farmers predominantly grow genetically modified (GM) hybrid maize. In small-scale systems, farmers rely on open pollinated varieties (OPVs) and/or landraces and are largely dependent on seed saving systems. The aim of this study was to understand the relationship between transgene expression rates, the resulting concentrations of the transgene product (Bt protein) and its bioactivity in insect pests following transgene flow from GM hybrid maize into non-genetically modified, non-GM near-isogenic maize hybrid (ISO) and OPVs. We modeled segregation patterns and measured *cry1Ab* transgene expression (mRNA quantification), Cry1Ab protein concentration and pest survival. Two groups of F1, F2 crosses and backcrosses with GM, ISO and OPV maize varieties from Brazil and South Africa were used. Bioassays with the larvae of two lepidopteran maize pest species, *Helicoverpa armigera* and *Spodoptera littoralis*, were carried out. Overall, the *cry1Ab* transgene outcrossed effectively into the genetic backgrounds tested. The *cry1Ab* transgene was stably expressed in both ISO and OPV genetic backgrounds. Transgene introgression led to consistent, though highly variable, concentrations of Cry1Ab toxins that were similar to those observed in GM parental maize. Most crosses, but not all, suggested the expected Mendelian segregation pattern. Transgene expression rates were significantly higher than expected from purely Mendelian segregation in the South African crosses. In South African materials, ISO and OPV crosses produced significantly lower Cry1Ab concentrations compared to the GM parental maize. The Cry1Ab toxins from crosses were bioactive and induced mortality rates of ≥92.19% in *H*. *armigera* and ≥40.63% in *S*. *littoralis* after a period of only 4 days. However, no correlations were observed between the quantitation of mRNA for *cry1Ab* and the corresponding Cry1Ab protein concentrations, nor between the Cry1Ab concentrations and insect mortality rates across different genetic backgrounds. We therefore suggest that while transcription of the *cry1Ab* transgene reliably determines the presence of Cry1Ab protein, mRNA levels do not reflect, by themselves, the end Cry1Ab protein concentrations found in the plant. Because predictably high Cry1Ab concentrations are a key prerequisite for effective insect resistance management (IRM) programs, this observation raises questions about the effectiveness of such programs in scenarios with complex crop genetic backgrounds. On the other hand, confirmed bioactivity in all crosses should be expected to impact small farmer’s selection behavior, unknowingly favoring the insecticidal trait. This may lead to a fixation of the trait in the wider population, and may influence the genetic diversity of varieties maintained by small-scale farmers.

## Introduction

Countries with economies in transition, such as Brazil and South Africa, are often characterized by a dual agrarian structure. Two types of farming systems may co-occur, sometimes side-by-side in the same region. While large-scale commercial farming systems produce crops for export as a commodity to global markets on the one hand, small-scale and subsistence farming largely supply to local markets where maize (*Zea mays)* is the prime staple crop on the other hand, and this is fundamental for rural food security. In the commercial systems, farmers predominantly grow genetically modified (GM, also known as transgenic) hybrid maize while in small-scale systems, farmers rely on open pollinated varieties (OPVs) and/or landraces outside of the hybrid maize seed economy. In the latter systems farmers depend on so-called "seed saving systems”, in which farmers select samples of seeds from their harvest for future planting. Landraces in this context are dynamic populations of maize which are genetically diverse and locally adapted [1], while OPVs are populations which were developed from landraces [2]. In Brazil and South Africa, GM maize hybrids in 2017 accounted for approximately 89% and 85% of the production, respectively. These GM maize hybrids carried transgenes for insect resistance, herbicide tolerance or the combination of both traits [3]. Resistance to pests is due to the expression of insecticidal gene toxins transferred into GM crops plants from the bacterium, *Bacillus thuringiensis* (Bt). These insecticidal proteins are produced throughout the growing season of the maize plants in leaf tissues, stalks, roots and pollen [4].

In order to delay the onset of target pest resistance to GM Bt maize, insect resistance management (IRM) strategies are deployed. These strategies require that Bt plants express the Bt toxin at a high dose, as well as the presence of a nearby adequate refuge area. In general terms, a high dose is described as the 25-fold dose necessary to kill 99% of susceptible pest individuals at the identical stage [5]. The refuge is composed of non-Bt maize plants to allow a sufficient number of homozygous susceptible pest individuals to survive and breed with potential heterozygous types emerging from the Bt-maize [5].

Maize is a wind-pollinated outcrossing plant with no known biological barriers to transgene flow from GM to non-GM plants (hybrids, OPVs and landraces) [6–12]. Therefore, the gene exchange between these types of maize becomes important, as does the unknown pattern of expression of transgenes introgressed into the less domesticated genetic backgrounds of OPVs and/or landraces and their resulting unknown Bt toxin concentration and variability. Lower concentrations of Bt toxins in F1 generation plants after transgene flow into non-GM maize have been documented [13]. This may influence exposure to Bt-toxins by the target pests shared between non-GM and GM maize, and their evolution of resistance against the insecticide [14, 15]. Furthermore, it is known that Bt protein concentrations in plants differ over time and in different tissues of plants [4, 16–19], opening the possibility that some of these concentrations may be too low to kill all heterozygote resistant pest individuals. Over the last years, despite the application of IRM practices in GM Bt crop fields, resistance evolution in target pest species has been reported around the world [20–28].

Evaluating the consequences of gene flow in general, regardless of whether or not transgenes are involved, is a challenge because it is difficult to predict the ecological and evolutionary effects of genes that are expressed in different ecological contexts and genetic backgrounds. Furthermore, the use of transgenes and their newly expressed proteins, such as insecticidal Bt toxins, allows the study of these processes in an elegant way, serving as markers both at the gene and protein level. Additionally, to determine Bt concentration and its variability in Bt hybrids as well as OPVs and landraces that have received the Bt transgene from commercial GM maize plants is crucial if impacts on resistance evolution in target pests is to be assessed. The occurrence of such introgressed Bt OPVs/landraces in small-scale farming systems may (pre-) expose and exert additional selection pressure on target pests to evolve resistance to Bt toxins. This, in turn, may lead unknowingly to accidental selection by farmers for the Bt trait in their maize varieties, which may have unpredictable consequences for the genetic diversity of their seeds. Thus, knowledge about the dynamics of transgene exchange is of fundamental importance to assess the sustainability of GM crop cultivation in compliance with the regulatory requirements of resistance management and biosafety, as well as from the perspective of small-scale farmers who may aim to cultivate and select GM-free OPVs and/or landraces seeds.

The scientific literature, documenting the entire three-way relationship of transgene expression, quantitative Bt production and bioactivity against target pests in outcrossed Bt x OPVs or other maize varieties is highly fragmented. Most studies have investigated two-way relationships, i.e. transgene expression–Bt protein production, or Bt protein production–bioactivity [13, 29–31]. Here, we report for the first time on the complete three-way relationship (Bt transgene expression, quantitative Bt toxin concentration and Bt toxin bioactivity against pest insects) in parent GM maize plants as well as in a range of outcrosses between GM, non-GM near-isogenic maize and OPVs from Brazil and South Africa. An additional aim of our study was to investigate segregation patterns of inheritance of transgenes.

## Material and methods

### Plant material, crosses and backcrosses: Brazil

The plant material used in this study was derived from seeds of GM maize (MON810) variety (AG5011YG), a non-GM near-isogenic maize variety (AG5011) and an OPV (Pixurum 5). The hybrid seeds were donated by the Agroceres Seeds Company from Brazil and the OPV seeds by Cooperative Oestebio of small-scale farmers from São Miguel do Oeste, Santa Catarina, Brazil. Verification of the GM-free status of the OPV seeds prior to the production of the crosses and backcrosses was done by means of ImmunoStrip^®^ enzyme-linked immunoassay tests (Agdia company, USA).

The GM hybrid used in this study (AG5011YG) is a three-way cross. Analysis showed that this transgene event had a single functional copy of the *cry1Ab* transgene coding sequence incorporated into the maize genome [32]. Additionally, other studies confirmed that the different MON810 maize varieties were hemizygous for the *cry1Ab* transgene [33–35]. The near-isogenic hybrid (AG5011) is also a three-way cross and adapted to the climatic growing conditions of the southern region of Brazil and differs from the variety AG5011YG only by the absence of the transgene. The Pixurum 5 is a stabilized OPV that has not been subjected to deliberate and intensive selection in a formal breeding program, but has been developed and conserved by small-scale farmers in Southern Brazil for around 20 years [36].

**Fig 1:**
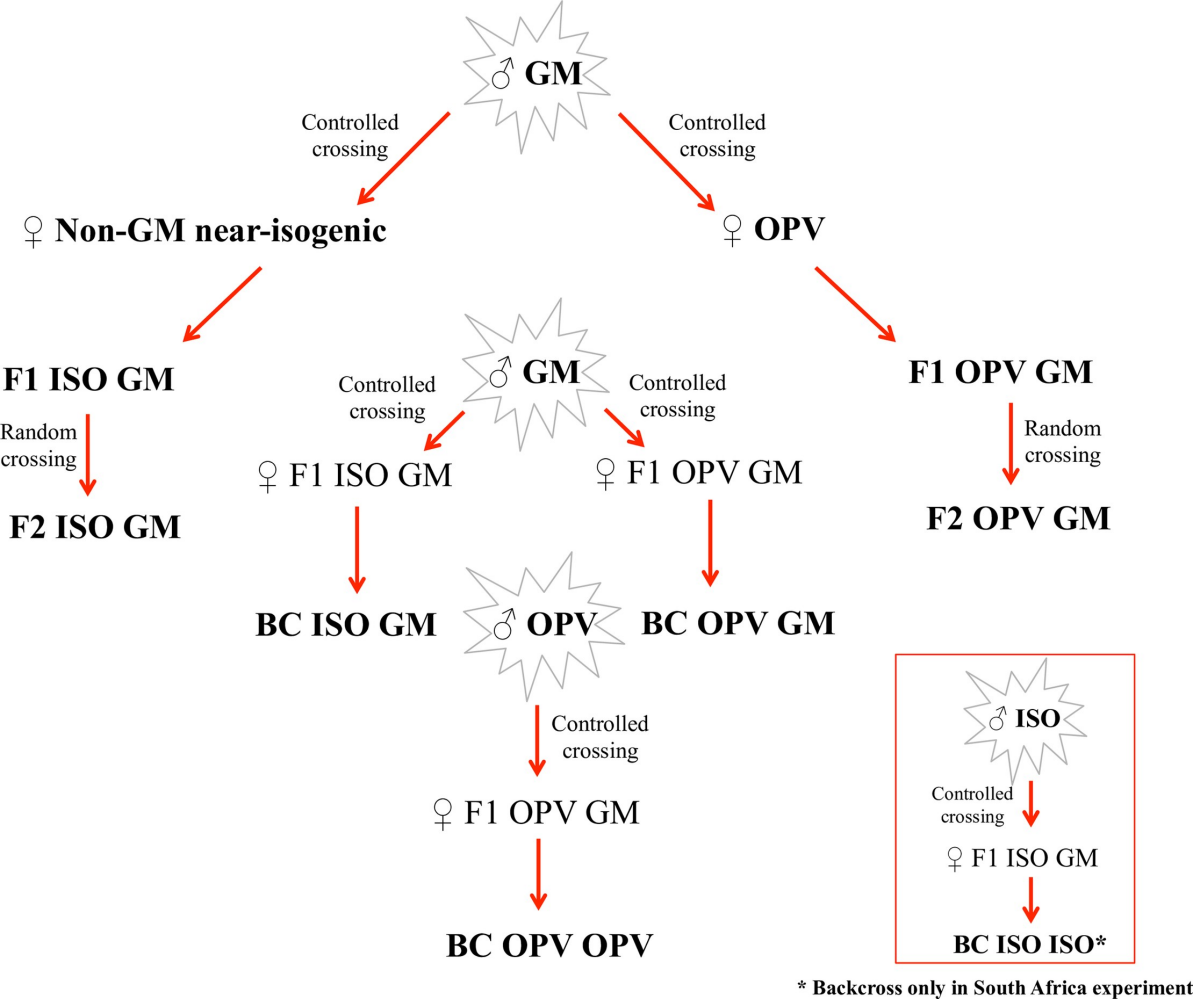
Diagram indicating how the crosses and backcrosses from Brazil and South Africa were obtained.

Crosses and successive backcrosses between GM (MON810/AG5011YG), non-GM near-isogenic maize (AG5011, called ISO here) and the OPV Pixurum 5 were generated under field conditions in Santa Catarina, Brazil. The development of the various crosses is described in Fig 1. The F1 crosses of OPV x GM maize and the ISO x GM maize were generated using the hybrid GM maize as male plant and the OPV and the non-GM maize as female, respectively. The segregating F2 populations (called F2 OPV GM and F2 ISO GM) were formed from the random crossing of the above-described F1 plants (Fig 1). The various back crosses (named BC) were produced by crossing the above-described F1 plants again with the male maize plants of their GM parents (BC OPV GM and BC ISO GM). The BC OPV OPV was produced by backcrossing F1 OPV GM plants with the male OPV parent maize again. In total, there were 3 parental maize plants (GM, non-GM near-isogenic and OPV), 2 F1’s (F1 ISO GM and F1 OPV GM), 2 F2’s (F2 ISO GM and F2 OPV GM) and 3 BC’s (BC ISO GM, BC OPV GM and BC OPV OPV) (Fig 1).

The F1 and BC populations were generated by growing the maize plants side by side in the field while removing the immature tassels (detasseling) of the female plants of interest to prevent natural fertilization with unwanted pollen. Maize plants were fertilized and irrigated according to local practices, and no pesticides or herbicides were used. Furthermore, the seedlings from all crosses and backcrosses were tested and only those with confirmed Bt protein expression were selected for the experiments.

### Plant material, crosses and backcrosses: South Africa

The plant material from South Africa consisted of seeds from GM maize (MON810) variety PAN 6Q 308B, non-GM near-isogenic maize variety PAN 6P-110 and an OPV (Kalahari). The crosses and backcrosses were done under field conditions. The way in which these crosses and backcrosses were generated is described in Fig 1. Compared with the crosses from Brazil, the difference was that here, in the latter, the F1 seedlings were tested for the presence of the Cry1Ab protein using Envirologix^®^ test strips. Seedlings that did not text positive were removed by hand, and only the Bt-positive seedlings were allowed to grow and were used for the subsequent backcrosses (BC) and F2’s. Although all offspring carried the transgene, expression of the Bt protein was also confirmed prior to the experiments.

#### Climate chamber conditions and ImmunoStrip^®^ tests

The GM parents, F1, F2 and BC maize seeds from Brazil and South Africa were planted in trays with 24 small pots filled with planting soil (Anzuchterde, Ökohum^®^, DE). To prevent Sciaridae flies from laying eggs in the soil, pots were covered with a layer of quartz fine gravel (fire-dried; Carlo Bernasconi AG, Switzerland). Seedlings were qualitatively tested for the presence of Cry1Ab protein using enzyme-linked immunoassay ImmunoStrip^®^ tests (Agdia^®^, USA), three weeks later. Only Bt-positive seedlings were transplanted into bigger pots (KREUWEL KC-Pflanzencontainer; V 4.4 L, ∅ 21 cm) filled with a potting soil (BioUniversalerde, Ökohum^®^, DE). The results of the presence/absence tests of the transgenic Cry1Ab protein in different genetic backgrounds from Brazil and South Africa are described in S1 and S2 Tables, respectively.

#### Experimental setup

All experiments were carried out over staggered time periods in the same climate chamber at the Swiss Polytechnic Institute (ETH) in Zürich. The experimental design was a randomized complete block. The experiments with the Brazil maize crosses were executed in four blocks and with 1–2 maize seedlings per different genetic background in each block. The majority of the different genetic backgrounds had eight maize seedlings each, but due to low germination rate and the limited number of seedlings with the GM trait in some of the crosses, the genetic background F1 ISO GM and BC OPV GM had only six maize seedlings each (S3 Table). In the experiments with the South African crosses, also executed in four blocks, the majority of the different genetic backgrounds had eight maize seedlings each but only the GM parental and the genetic background F1 ISO GM had seven seedlings (S3 Table). During germination and growth, the seedlings were arranged randomly in a climate chamber (Kälte 3000 AG) with regular watering and daily 16h light, 25°C and 50% rh - 8h dark, 20°C and 65% rh. The technical light information of the climate chamber was: 100% ~ 28 klux ~ 356 PPDF [μmol.m-2.s-1] 1m above plants.

### Plant sampling

After two weeks after planting, plants were sampled to determine transgene expression, Cry1Ab concentration and to conduct bioassays. At that time, the maize plants were between the V5 and V7 stages and, for each plant, the fifth fully developed leaf was sampled. Six circular leaf samples (∅ 1.5 cm) were cut out with a cork-cutter, three on each side of the central part of the leaf but avoiding the main leaf vein (S1 Fig) [37]. The leaf disks assigned for the transgene expression analysis were immediately frozen in liquid nitrogen and later stored at -80°C. The leaf disks designated for quantification of the Cry1Ab concentration were kept on ice at -20°C during transport to storage the same day. Leaf pieces designated for the bioassay feeding trials with the two lepidopteran species were used immediately for the trials that were started on the same day that samples were taken.

### qRT-PCR

Reverse transcriptase, real-time polymerase chain reaction assays (qRT-PCR) were used to establish a proxi for gene transcription activity in plant samples.

Following the protocol method of the RNA plant NucleoSpin^®^ kit (Macherey-Nagel, DE), RNA was extracted from 52 maize leaf samples from the Brazilian crosses (at least 6 samples per different genetic background) and from 70 maize leaf samples from the South African crosses (at least 7 samples per different genetic background). The RNA concentration in extracts was determined with a Qubit^®^ fluorometer (Invitrogen™, USA) and their purity checked on Agilent 2200 TapeStation System. Afterwards, the RNA was treated with RDD Buffer (Qiagen^®^, DE) and DNAse (Qiagen^®^, DE) and inactivated with RNase-free H_2_O. All assays were run with a standardized total RNA concentration of 4 μg/ml.

The cDNA synthesis was carried out using the QuantiTect^®^ Reverse Transcription Kit (Qiagen^®^, DE), including the Wipeout Buffer and RT Primer mix that contains a specially optimized mix of oligo-dT and random primers to enable cDNA synthesis from all regions of RNA transcripts, even from 5’ regions. Each maize sample was run in triplicate in a reaction volume of 10 μl using TaqMan^®^ Gene Expression Master Mix (Applied Biosystems^®^, USA) and 1 μl of cDNA (S4 Table). The instrument used in the analysis was the ABI 7500 FastRT-PCR (7500 Software v2.0) from Applied Biosystems^®^, USA.

The primers and probe sequences for *cry1Ab* transgene were kindly provided by A. Coll (Institut de Tecnologia Agroalimentària (L’INTEA), Universitat de Girona). Additionally, three reference genes (*mep*, *ubcp*, *lug*), as recommended by Manoli et al. [38], were chosen to normalize the qRT-PCR data. TaqMan primers and probes for reference genes were designed based on the sequences obtained from Maize Genetics and Genomics Database (http://www.maizegdb.org/) using Primer Express 3.0 software (Applied Biosystems, USA).

### Enzyme-linked immunosorbent assay (ELISA)

Enzyme-Linked Immunosorbent Assay (ELISA) was used to quantify the Cry1Ab concentration in the same leaf samples that were also used for the transgene expression (RT-qPCR) analyses. This allowed the simultaneous determination of Bt concentration and mRNA as a proxi of transgene activity. Between 5 to 10 mg of freeze-dried leaf material was ground using a FastPrep-24 Instrument (MP Biomedicals, Inc.) and homogenized in 1.5 ml of PBST-buffer (pH 7.4). After centrifugation the supernatants were diluted 1:100 with PBST-buffer. Standards were prepared using freeze-dried Cry1Ab toxin (M. Pusztai-Carey, Case Western Reserve University) similar to the Cry1Ab protein expressed in the MON810 maize plants. Twelve Cry1Ab concentrations were used for the calibration curve ranging from 0 to 4.4 ng/ml dissolved in PBST-buffer. The Cry1Ab concentration in the different genetic backgrounds was determined using the commercial double antibody sandwich (DAS) ELISA kit (Agdia^®^, USA). The standards were added to a 96-well ELISA microplate in duplicates, and the negative controls and the samples were added in triplicates. The optical density development was measured on a SPARK 10M multimode microplate reader (TECAN^®^) at 650 nm.

### Bioassays

#### Insects

The lepidopteran pests used in this study were *Helicoverpa armigera* (Noctuidae) and *Spodoptera littoralis* (Noctuidae). Eggs of *H*. *armigera* were obtained from ENTOMOS AG (Switzerland) and kept in a growth chamber (Sanyo MLR 350) at 18°C, at photoperiod of 16:8 h (L:D) and light intensity of 20% (the type of the fluorescent lamp of growth chamber was FL40SS W/37). Larvae were reared in the laboratory on a wheat-germ based artificial diet [39] until the start of the bioassays with second-instar larvae. The diet contained the following ingredients: deionized water, agar powder, organic corn semolina, wheat germ, yeast powder, benzoic acid, nipagin and ascorbic acid.

Additionally, second-instar larvae of *S*. *littoralis* were provided by Syngenta Crop Protection (Stein, Switzerland).

According to the scientific literature, *H*. *armigera* and *S*. *littoralis* are considered non-target insect pests of GM maize plants that express the Cry1Ab protein because of their medium susceptibility to this Bt protein which does not allow for satisfactory crop protection [40–42]. In our trials, however, this was desired in order to test for a dose-dependent response, which is much less possible in highly susceptible species.

#### Mortality bioassays

The bioactivity of the Cry1Ab protein was measured in mortality bioassays conducted with second-instar larvae. In the bioassays, *H*. *armigera* were fed with leaf tissue from the Brazilian crosses, while *S*. *littoralis* were fed with leaf tissue from the South African crosses. Thirty-two-well trays were used. In each well, a blotting paper (2 cm x 2 cm) was placed and moistened with distilled water to maintain sufficient humidity. A piece of the fresh leaf test material, sampled simultaneously with the leaf test material sampled for the RNA extraction and ELISAs, was added together with one larva of either *H*. *armigera* or *S*. *littoralis*. Finally, the trays were closed with adhesive transparent lids with small perforations to allow for air circulation. The trays were placed in a climate chamber at 26°C, a photoperiod of 16:8 h (L:D) and light intensity of 20% (same lighting conditions as described before). Eight wells were designated to one maize plant, or one sampled leaf (S2 Fig). In total, the Brazilian crosses were tested in four blocks each, using tissue from six maize leaves from each of the F1 ISO GM and BC OPV GM crosses, respectively, and eight maize leaves from the other genetic backgrounds. The South African crosses were tested in four blocks each, using tissue from seven maize leaves each from the GM parent and F1 ISO GM plants, respectively, and also tissue from eight maize leaves each from the other genetic backgrounds. The number of live larvae was determined 4 days after the bioassays commenced. The trays with the larvae inside were placed at -20°C and later disposed of.

### Statistics

#### Segregation pattern analysis—Pearson’s chi-squared test (χ^2^ test)

Pearson’s chi-square (χ^2^) test [43], was applied when there was one nominal variable with two or more values (such as positive and negative Bt maize plants). Observed counts of each category were compared with the expected counts, calculated on the theoretical expectation that they would follow Mendel’s law, which produces specific ratios of positive and negative Bt maize plants after crosses. This method is supported by precedent in many other crop studies, e.g. in maize [44, 45], in rice [46], triticale [47] and tomato [48].

Positive / negative scores for plants were determined using lateral flow ELISA tests (see above) appropriate for qualitative testing of presence or absence of the Bt proteins. These ratios were compared to the expected Mendelian inheritance ratio by using the following equation:

$$\chi^{2}=\frac{\sum {\left(\mathrm{observed}-\exp\mathrm{ected}\right)}^{2}}{\exp\mathrm{ected}}$$

For example, in a random sample of 100 maize seedlings in F1 OPV GM, following Mendel’s law, the expectation for positive and negative Bt maize plants are equal in frequency (50:50). Therefore, in the genetic background of our study, the observed number of Bt-positive (n = 38) and Bt-negative (n = 62) seedlings was compared to the theoretical frequencies of 50 positive and 50 negative Bt maize plants, using the following equation:

$$\chi^{2}=\frac{{\left(38-50\right)}^{2}}{50}+\frac{{\left(62-50\right)}^{2}}{50}=5.76$$

Subsequently, the calculated χ^2^ was compared with the tabulated χ^2^, at a significance level of 0.05 and one degree of freedom, with results in the tabulated value of 3.84. The degree of freedom (n-1) is based on the number of categories tested in the genetic background (n) (i.e. Bt-positive and Bt-negative), in this case, two *minus* one, yielding one degree of freedom.

#### Transgene expression—mRNA quantitation

The threshold cycles (Ct) of the transcripts in the samples were calculated by means of Real-time PCR software and data were exported to Microsoft Excel. In cases where the Ct standard deviation for the triplicate group exceeded the default setting of the instrument (0.5), or in the presence of outliers, the samples were used in duplicate. Amplification efficiencies of all crosses were estimated using LinRegPCR software version 2012.3 [49] and the values are presented in the S5 and S6 Tables, respectively. The stability of the three reference genes was assessed using geNorm (Excel) and for normalization (M < 0.5 and pair-wise coefficient variance < 0.15) of the mRNA quantitation data (as a proxi for transgene expression) using the qbase+ software (Biogazelle^®^). The qbase+ program facilitates the process of validating reference genes and performing state-of-the-art normalization using the geometric mean of multiple validated reference genes.

A two-way ANOVA was performed to evaluate the significance of the effects of different genetic backgrounds and different blocks on the mRNA levels as a measure of transgene expression levels. However, outliers and the interaction between different genetic backgrounds and different blocks were excluded from the analyses.

#### Cry1Ab concentration

Cry1Ab concentration was calculated by using a linear regression equation for the standard curve including only triplicate samples with coefficient of variation less than 20%. Cry1Ab concentrations in maize leaves from different genetic backgrounds were expressed in μg/g dwt (dry weight tissue).

Additionally, homogeneity of variances in Cry1Ab concentration between the different genetic backgrounds and groups was calculated using the Fligner-Killeen test.

#### Mortality bioassays

Larval mortality was recorded four days after the onset of the experiments. A General Linear Model (Binomial method) was used to analyze the effects of different genetic backgrounds on insect mortality rate.

#### Correlation analyses

Due to non-normal distribution in the data, the correlation between transgene mRNA expression and Cry1Ab protein, and the mortality rate and Cry1Ab protein were calculated using the Spearman rank correlation coefficient (*Rs*).

#### Computational analyses

Analyses were conducted in R [50] and figures were produced using the package ggplot2 [51]. For multiple comparisons analyses the package multicomp [52] was used and for pairwise comparisons the package emmeans was used [53].

## Results and discussion

### Segregation patterns in different genetic backgrounds

Most crosses followed an expected Mendelian segregation pattern. Among the Brazilian crosses, F2 OPV GM and BC ISO GM exhibited Mendelian inheritance of the *cry1Ab* transgene as detected by positive signals in ELISA tests for Cry1Ab protein at a significance level of < 0.05. F1 ISO GM and F1 OPV GM also showed Mendelian segregation at a significance level of < 0.01. In contrast, the genetic backgrounds BC OPV OPV and BC OPV GM deviated greatly from the expected Mendelian inheritance, with strikingly fewer plants showing a positive signal (protein detected) of the GM trait than expected (S1 Table). Among the South African crosses, except for F2 ISO GM, all crosses showed Mendelian inheritance at a significance level of 0.05. However, when analyzed at a significance level of 0.01, all different genetic backgrounds matched the expected Mendelian pattern (S2 Table).

Crosses F1 ISO GM and F1 OPV GM showed some Bt-negative maize plants, suggesting that the GM parental varieties used in this study carried only a single copy of the transgene, since all F1 maize plants would have been positive for the presence of the transgene if the parent plants had two or more copies of the transgene, assuming the Mendelian expectation. This suggestion is consistent with previous studies on MON810 maize [33–35] as well as a technical report from the original producer of the parental varieties [32]. This observation suggests the validity of our modeling approach to establish segregation behavior in our crosses.

Many studies report that the mode of transgene inheritance follows directly a Mendelian segregation pattern [54–57] In maize, Duncan et al. [58] reported that after GM traits were introgressed into landrace maize varieties the transgene segregated like other endogenous genes, following Mendelian principles,. In contrast, non-Mendelian segregation of transgenes has been reported in studies on rice [59], soybean [60], tobacco [61], wheat [62] and cotton [63, 64]. In a study with white clover, non-Mendelian segregation was found in F2 populations and the distortion was influenced by genetic background [65]. A mix of both Mendelian and non-Mendelian segregation of transgenes has been observed in studies with rice [66], maize [67], castor [68] and wheat [69]. Unpredictable inheritance of transgenes has been variously attributed to silencing or transgene instability [65, 66]. Factors cited to influence apparent segregation of the transgene trait include the nature of the recipient genome (genetic background, gamete viability, chromosome abnormality, transformation method), the nature of the transgene itself (transgene silencing and unstable integration of the transgene) and the interaction between these two (homozygous lethality, poor transmission of the transgene and mitotic crossover/meiotic instability) [70]. Based on the qualitative analysis presented here, it is not possible to confirm which mechanisms influenced the cases where distortion in Mendelian segregation was observed. This we have to leave for future research projects to take on.

Since no Bt protein was detected in the BC OPV OPV cross, this backcross had to be excluded from further analyses. Many factors could have contributed to the absence of the GM trait in this backcross.

Independently of the mechanism involved, non-mendelian transgene trait behavior can have important practical implications. Stable expression over many generations is inherent to the sustainability of the transgenic method itself [71], but a range of other consequences derive from deviations happening in crosses between GM and non-GM maize plants (hybrids, OPV’s and landraces varieties) under field conditions. This is especially significant in maize populations maintained by small-scale farmers. Thus, for example, enhanced fitness in the hybrid progeny conferred by the insect-resistance transgene could alter the seed selection behavior of small-scale farmers who, consequently, would inadvertently exert negative selection against non-GM maize materials. In addition, it is evident that transgenes conferring strong selection advantage, such as insect-resistance, to the carrier plant *per se* may have evolutionary impacts on the hybrid progeny by changing patterns of allelic segregation after introgression from GM to non-GM crops [72]. These factors should be taken into consideration when assessing the long-term environmental impacts of transgene introgression into GM-free populations.

### Transgene expression

The measurement of specific mRNA products, via the RT-qPCR method, was considered a reasonable proxi for actual transcription rates of the *cry1Ab* transgene, i.e. transgene expression [73].

The mean transgene transcription level in the leaves of different genetic backgrounds ranged between 0.75 in BC ISO GM and 1.64 in F1 ISO GM in the Brazilian crosses. Mean expression, in mRNA levels, in leaves of the South African crosses was 0.70 in BC OPV OPV and 1.45 in F2 OPV GM (Table 1).

**Table 1:** Number of analyzed maize plants, mean and standard error (±SE) of the relative transgene expression in different genetic backgrounds from Brazil and South Africa.

Genetic background	N° of plants analyzed		Rel. transgene expression ± SE	
	Brazil	South Africa	Brazil*	South Africa**
GM	8	6	1.57 ± 0.23	1.25 ± 0.13
F1 ISO GM	5	7	1.64 ± 0.16	0.90 ± 0.19
F2 ISO GM	8	8	1.35 ± 0.19	1.29 ± 0.21
BC ISO GM	7	8	0.75 ± 0.03	1.21 ± 0.19
BC ISO ISO	-	8	-	0.78 ± 0.17
F1 OPV GM	8	8	1.02 ± 0.18	1.03 ± 0.14
F2 OPV GM	8	6	1.12 ± 0.21	1.45 ± 0.28
BC OPV GM	6	8	1.30 ± 0.30	0.79 ± 0.18
BC OPV OPV	-	8	-	0.70 ± 0.09

* *F* = 2.196; df = 6; *P* = 0.063.

** *F* = 2.141; df = 8; *P* < 0.05.

**Fig 2:**
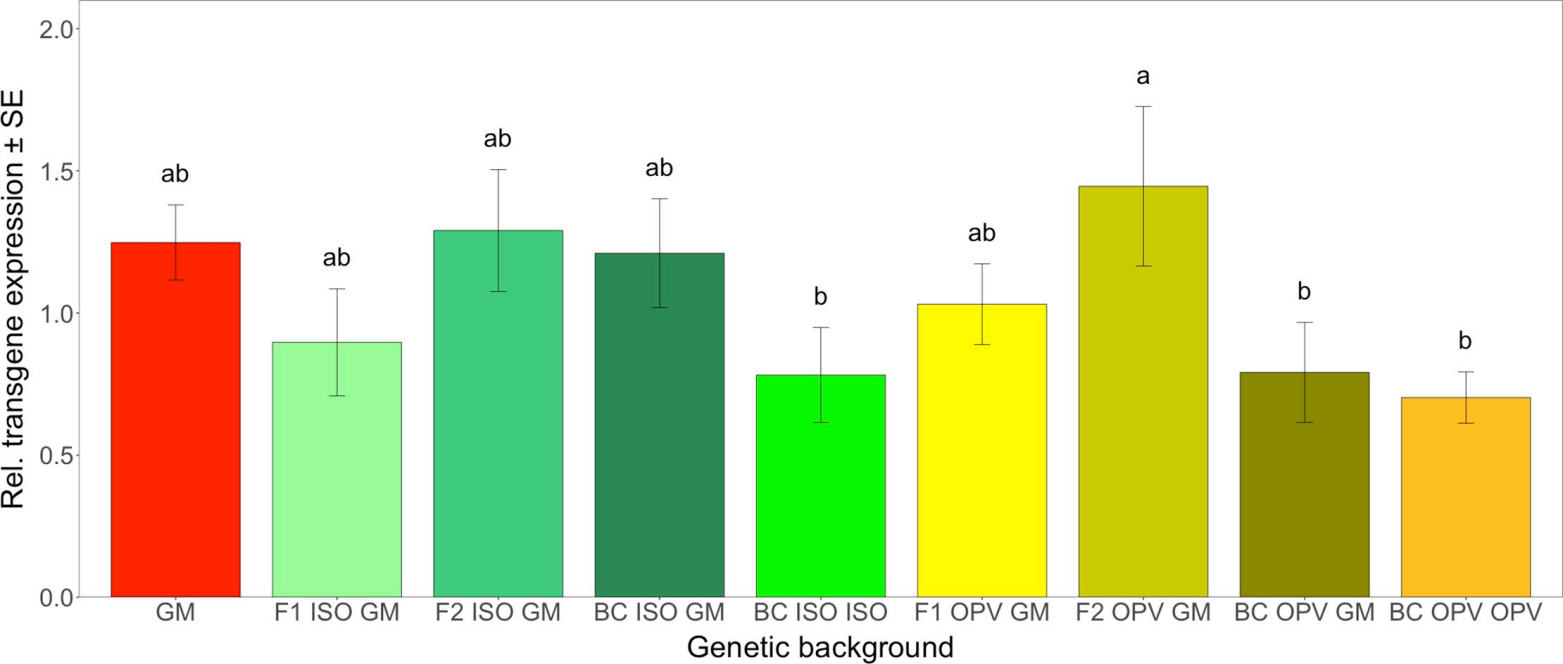
Mean transgene expression (transcription activity) in different genetic backgrounds from South Africa. Vertical bars show mean values, with the standard error (± SE) indicated as lines. Letters above the bars represent results from the means separation Duncan’s test.

In the Brazilian crosses, there was no significant difference in relative transgene transcriptional activity between the different genetic backgrounds (*F* = 2.196; df = 6; *P* = 0.063). However, in the South African crosses, there was a significant difference in the relative transgene expression—as expressed in mRNA levels—between different genetic backgrounds (*F* = 2.141; df = 8; *P* < 0.05). Hence, a means separation test (Duncan’s test) was applied and the results are shown in Fig 2. In the South African crosses, transgene transcriptional levels were significantly higher in the genetic background F2 OPV GM (1.45) than in BC ISO ISO (0.78), BC OPV GM (0.79) and BC OPV OPV (0.70). Results also showed that the *cry1Ab* transgene was transcribed at similar levels in different genetic backgrounds in both groups of crosses (Brazilian and South African) compared to the respective GM parental maize. These results also demonstrated that, except for the one case mentioned above (F2 OPV GM), there was no clear evidence for a significant increase of the mean transgene activity level due to the formation of homozygous maize plants carrying two copies of the transgene in the F2’s and BC’s. However, because no direct assessment of zigosity was attempted, this must remain an indirect indication. In any case, there have been mixed results when investigating the effect of gene dosage (including through zygosity) and transgene expression [74, 75].

In order to highlight differences between larger groups, different genetic backgrounds were pooled according to ISO or OPV parental lines, without differentiation between F1’s, F2’s and BC’s. These pooled groups were renamed ISO crosses and OPV crosses, and compared. Results from this comparison are shown in Table 2.

**Table 2:** Number of maize plants analyzed, mean and standard error (±SE) of relative transgene transcription activity in different groups from Brazil and South Africa.

Group	N° of plants analyzed		Rel. transgene expression ± SE	
	Brazil	South Africa	Brazil*	South Africa**
GM	8	6	1.57 ± 0.24	1.25 ± 0.13
ISO crosses	20	31	1.21 ± 0.12	1.05 ± 0.10
OPV crosses	22	30	1.13 ± 0.13	0.96 ± 0.10

* *F* = 2.133; df = 2; *P* = 0.131.

** *F* = 0.788; df = 2; *P* = 0.459.

There was no significant difference in mean transgene transcription levels between the different groups from Brazil (*F* = 2.133; df = 2; *P* = 0.131) and South Africa (*F* = 0.788; df = 2; *P* = 0.459), according to the ANOVA test. In sum, no significant difference was observed in the transgene transcription levels when inserted into homogeneous and heterogeneous genomic backgrounds (i.e. ISO crosses versus OPV crosses), when compared to GM parental maize.

Overall, the data demonstrated that the *cry1Ab* transgene expression was stable after transgene flow into different genetic backgrounds in both groups of crosses from Brazil and South Africa. Moreover, there was no significant difference in transgene expression, as measured by mRNA quantitation, between homogeneous and heterogeneous genomic backgrounds (i.e. ISO crosses versus OPV crosses).

### Cry1Ab concentration

The mean concentration of Cry1Ab protein in the leaves of different genetic backgrounds ranged between 35.12 (μg/g dwt) in F2 ISO GM and 48.48 in F2 OPV GM in the Brazil group of crosses, and 20.40 in F1 OPV GM and 40.85 in GM parental maize in the South African group of crosses (Table 3).

**Table 3:** Number of maize plants analyzed, mean and standard error (±SE) of the Cry1Ab concentration in the different genetic backgrounds from Brazil and South Africa.

Genetic background	N° of plants analyzed		Cry1Ab concentration (μg/g dwt) ± SE	
	Brazil	South Africa	Brazil*	South Africa**
GM	8	6	42.78 ± 5.68	40.85 ± 4.57
F1 ISO GM	5	7	48.26 ± 9.00	27.47 ± 5.05
F2 ISO GM	6	8	35.12 ± 6.21	24.45 ± 4.17
BC ISO GM	8	7	45.59 ± 6.74	30.54 ± 4.60
BC ISO ISO	-	8	-	28.18 ± 4.10
F1 OPV GM	7	8	41.27 ± 3.97	20.40 ± 2.90
F2 OPV GM	8	8	48.48 ± 4.87	28.35 ± 5.39
BC OPV GM	5	8	39.78 ± 9.24	25.39 ± 3.53
BC OPV OPV	-	8	-	25.35 ± 2.17

* *F* = 0.628; df = 6; *P* = 0.707.

** *F* = 1.453; df = 8; *P* = 0.196.

There was no significant difference in Cry1Ab concentration in plant tissues between the different genetic backgrounds from Brazil (*F* = 0.628; df = 6; *P* = 0.707) and from South Africa (*F* = 1.453; df = 8; *P* = 0.196). In other words, no signal could be detected relating possible gene-dosage effects to Cry1Ab concentration. This finding stands in contrast with previous reports suggesting that homozygous GM maize hybrids had increased expression levels of Bt proteins and increased *S*. *frugiperda* control [76].

In order to highlight differences between the main genetic backgrounds, data were pooled according to ISO or OPV parental lines, without reference to F1’s, F2’s and BC’s. The pooled groups were renamed as ISO crosses and OPV crosses and the comparison between them in terms of Cry1Ab protein concentration described in Table 4.

**Table 4:** Number of maize plants analyzed, mean and standard error (±SE) of the Cry1Ab protein concentration in the main genetic background groups from Brazil and South Africa.

Group	N° of plants analyzed		Cry1Ab concentration (μg/g dwt) ± SE	
	Brazil	South Africa	Brazil*	South Africa**
GM	8	6	42.78 ± 5.68	40.85 ± 4.57
ISO crosses	19	30	42.98 ± 4.15	27.57 ± 2.15
OPV crosses	20	30	43.78 ± 3.24	25.17 ± 1.88

* *F* = 0.058; df = 2; *P* = 0.943.

** *F* = 5.443; df = 2; *P* <0.05.

**Fig 3:**
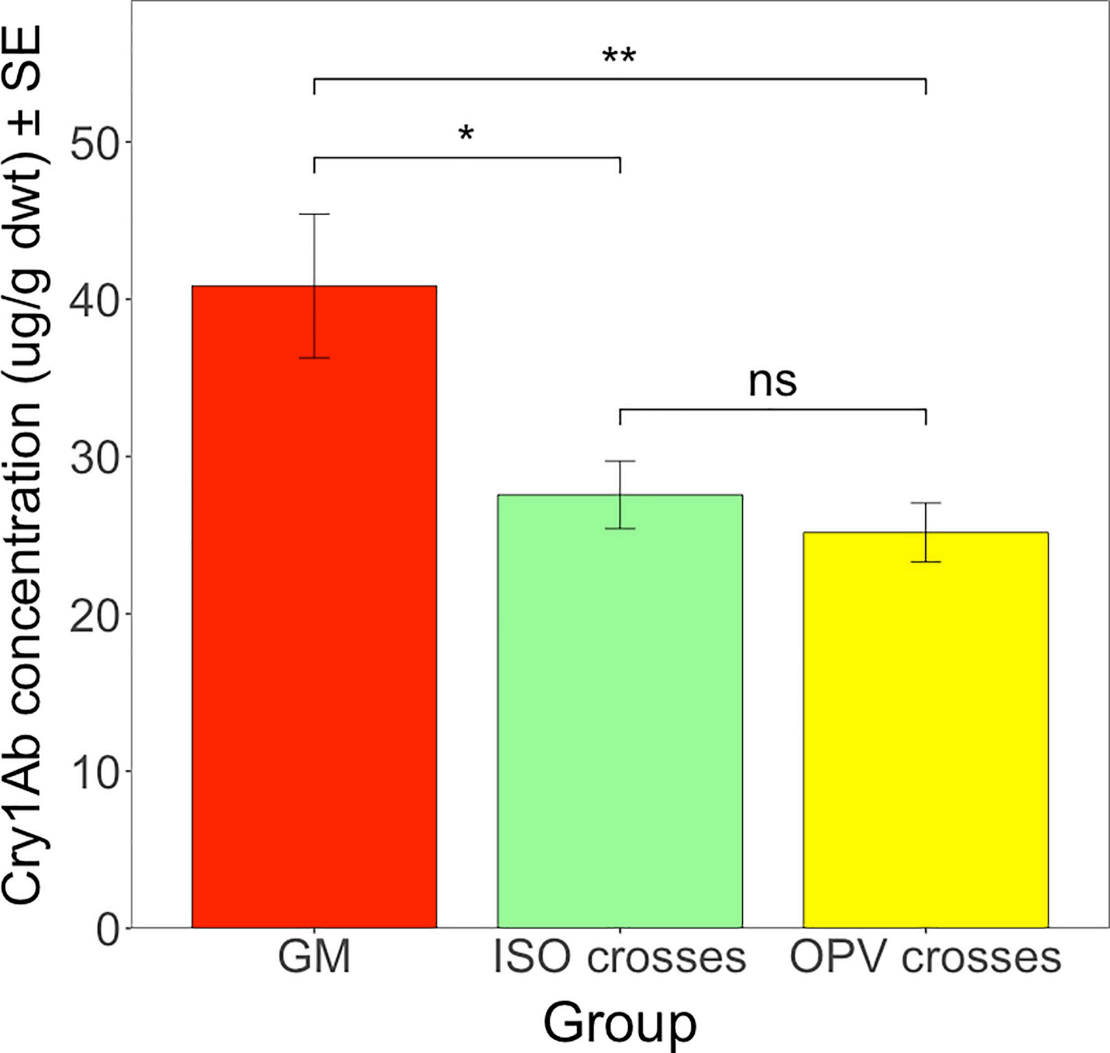
Mean Cry1Ab concentration (μg/g dwt) in different groups from South Africa. Vertical bars show mean values, with standard error (± SE) indicated as lines. * *P* < 0.05, ** *P* < 0.01, ns = not significant.

Again, there was no significant difference in Cry1Ab concentration between the different groups of crosses from Brazil (*F* = 0.058; df = 2; *P* = 0.943). We take this to imply that the *cry1Ab* transgene was equally transcribed and translated in both the homogenous genome (ISO crosses) and the heterogeneous genome (OPV crosses). However, there was a significant difference in Cry1Ab concentration between the different groups from South Africa (*F* = 5.443; df = 2; *P* < 0.05). The pairwise comparisons between the different crosses from South Africa revealed a significant difference in the Cry1Ab concentration between the GM parental maize and the ISO crosses (*t* ratio = 2.746; df = 62; *P* < 0.05) and between the GM parental maize and the OPV crosses (*t* ratio = 3.299; df = 62; *P* < 0.01). The analysis did not show a significant difference between ISO crosses and OPV crosses (*t* ratio = 0.958; df = 62; *P* = 0.606) (Fig 3).

Likewise, no significant differences in variances for Cry1Ab concentration were observed in the Fligner-Killeen test between different genetic backgrounds from Brazil (*P* = 0.821) or South Africa (*P* = 0.449) nor between the different crosses from Brazil (*P* = 0.446) or South Africa (*P* = 0.831). These results show that the measured Cry1Ab concentrations in the different genetic backgrounds and crosses tested are highly variable, but that this variability was similar in all backgrounds even when compared to the Bt toxin concentrations measured in the GM parental maize varieties.

It is relevant to compare results of this study with another study carried out in partnership to our research project, using the same genetic backgrounds from South Africa but under fluctuating greenhouse conditions [13]. Under those conditions, the F1 OPV GM genetic background produced significantly lower Cry1Ab protein concentrations (8.9 μg/g dwt) compared to the GM parental maize and other genetic backgrounds [13]. However, their F2 ISO GM and BC ISO GM crosses produced concentrations of 23.1 and 24.0 μg/g dwt, respectively, that were significantly higher compared to GM parental maize (14.9 μg/g dwt) [13]. The implications of lower Cry1Ab concentrations in these two groups of genetic backgrounds from South Africa, after (unnoticed) gene flow from GM maize into non-GM maize (hybrid and/or OPV), could accelerate the rate of insect resistance evolution in small-scale farming systems.

### Correlation between transgene transcription levels and Cry1Ab concentration

In this study we were able to obtain matching data for three levels of transgene expression for each and every sample considered: transcription into mRNA, protein synthesis and bioactivity against two insect herbivores. This opens the unique opportunity to analyze these values in their correlative relationship, in order to suggest specific areas of attention for further research and application of transgene-based agronomic practices. We therefore consider below two kinds of correlation between each of the three levels of transgene expression: transcription vs. protein concentration, and protein concentration vs. bioactivity against herbivore insect species. In addition, we provide a contrast between the effects of the transgene expression on the two insect species utilized.

**Fig 4:**
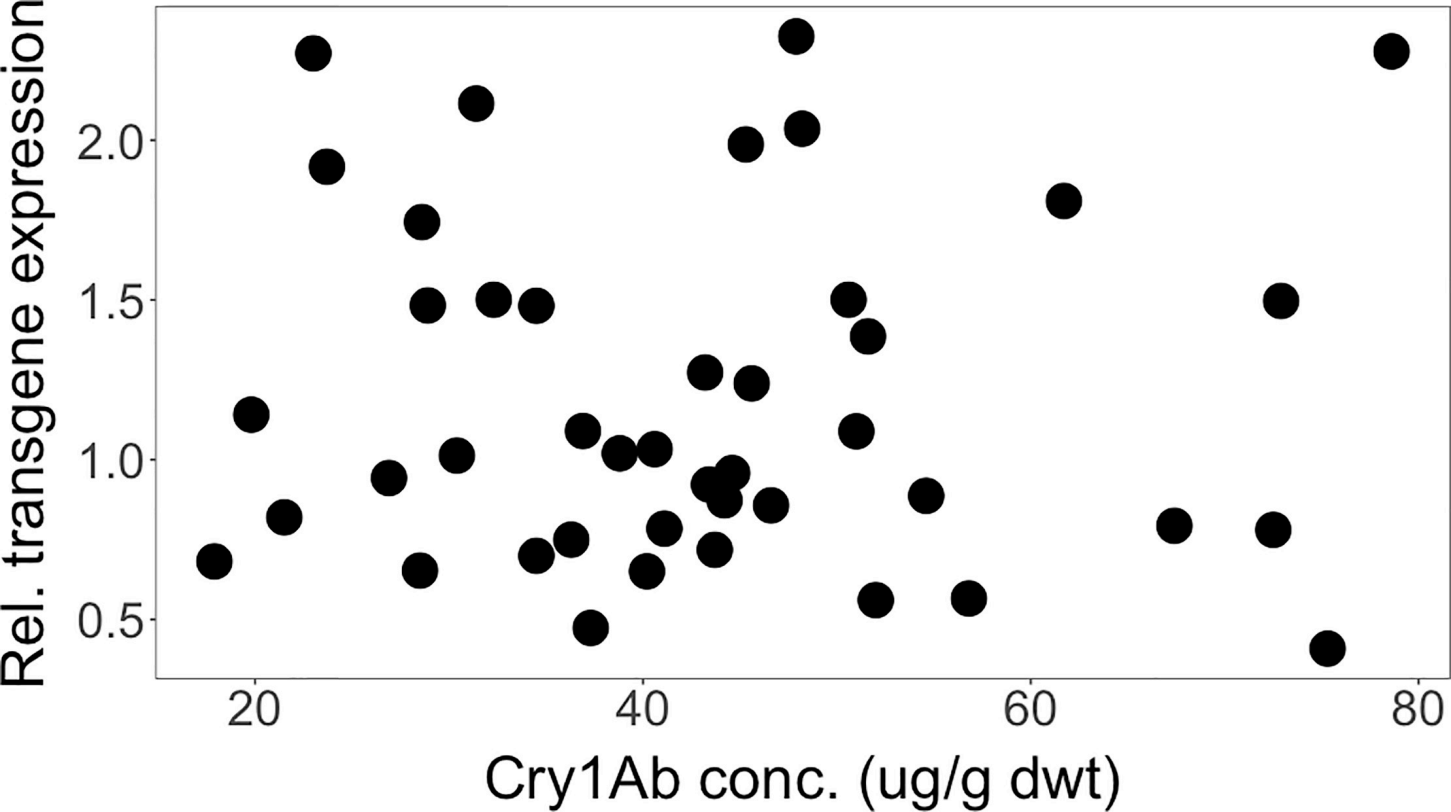
Spearman rank correlation between relative transgene expression and Cry1Ab concentration across different genetic backgrounds from Brazil. (*Rs* = -0.03, *P* = 0.830).

**Fig 5:**
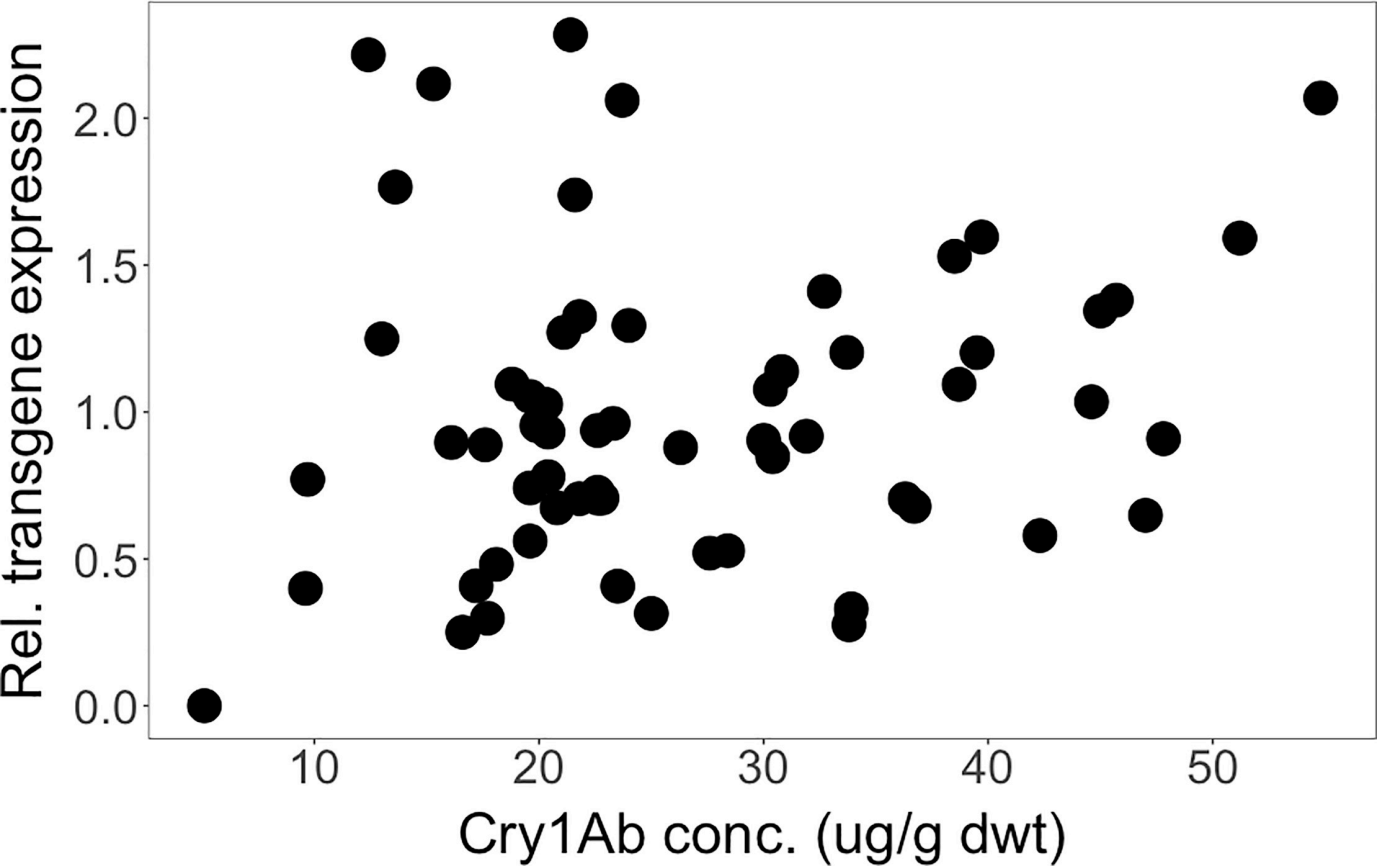
Spearman rank correlation between relative transgene expression and Cry1Ab concentration across different genetic backgrounds from South Africa. (*Rs* = 0.169, *P* = 0.190).

We found no statistically significant correlation between the Cry1Ab concentrations and the transgene transcription rates, measured as mRNA relative concentrations, across different genetic backgrounds from Brazil (*Rs* = -0.03, *P* = 0.830) (Fig 4) or from South Africa (*Rs* = 0.169, *P* = 0.190) (Fig 5). This means that the transcription rates of the transgene had little impact on the concentration of the Cry1Ab toxin ultimately produced. In the Brazilian crosses, concentrations of Cry1Ab toxin differed between around 25 μg/g dwt and almost 80 μg/g dwt–a more than 3-fold difference, even though transgene expression rates were similarly high (ca. 2.0–2.3). *Vice versa*, highest Cry1Ab concentrations occurred at transgene expression rates of as low as 0.4 and as high as 2.3 –a more than 5-fold difference. In the South African crosses, the highest transgene expression rates (ca. 2.0–2.3) yielded concentrations of Cry1Ab toxin that varied between 10 μg/g dwt and 60 μg/g dwt–a roughly 6-fold difference. And, *vice versa*, highest Cry1Ab concentrations occurred at transgene expression rates from around 0.5 to 2.0 –a roughly 4-fold difference.

When separating different genetic backgrounds, results showed no correlation between Cry1Ab concentrations and transgene expression rates in any genetic background from Brazil (S7 Table). However, in the South African crosses Cry1Ab concentration was correlated with *cry1Ab* transgene expression rate at least in the BC ISO GM (*Rs* = 0.88, *P* = 0.007) and the F2 OPV GM crosses (*Rs* = 0.74, *P* = 0.046). In other genetic backgrounds from South Africa, results also failed to reveal any correlation (S7 Table).

Additionally, when grouping data according to ISO or OPV genetic backgrounds, there was no correlation between Cry1Ab concentrations and transgene expression rates in any of the crosses from Brazil or South Africa (S8 Table).

These results lie in contrast with a separate study using GM maize seeds from South Africa (not crossed into heterogeneous backgrounds), where we did find differential values between hybrids regarding *cry1Ab* transgene expression and Bt protein concentrations, with a positive correlation for one hybrid [31]. Ponnala et al. [77] also showed a positive correlation between transcript levels and corresponding proteins in maize, in their case using non-GM varieties. In other GM crops, others have reported a correlation between transgene transcription and Cry1Ab concentrations for transgenic tomato [48, 78] and cotton [29, 30]. We are led to state, as a working hypothesis, that the crossing of the *cry1Ab* transgene into heterogeneous genetic contexts differs significantly from crossings within well-defined homogenous backgrounds.

Our results document that the mRNA levels of the *cry1Ab* transgene do not appear to directly determine in any measurable way the concentration of the produced Cry1Ab toxin. We suggest that there may be other plant regulatory processes influencing the ultimate concentration levels of the Cry1Ab toxin, such as post-transcriptional, translational and protein degradation/synthesis regulation, or even variation in promoter activity [29, 79–81] related to the heterogeneous context surrounding the transgene. In any case, our results clearly show that transgene product concentration (Cry1Ab protein) appears to be unpredictable from a measurement of the transgene transcriptional process in the specific, heterogeneous genetic contexts we studied.

### Mortality rates of *H*. *armigera* and *S*. *littoralis*

**Fig 6:**
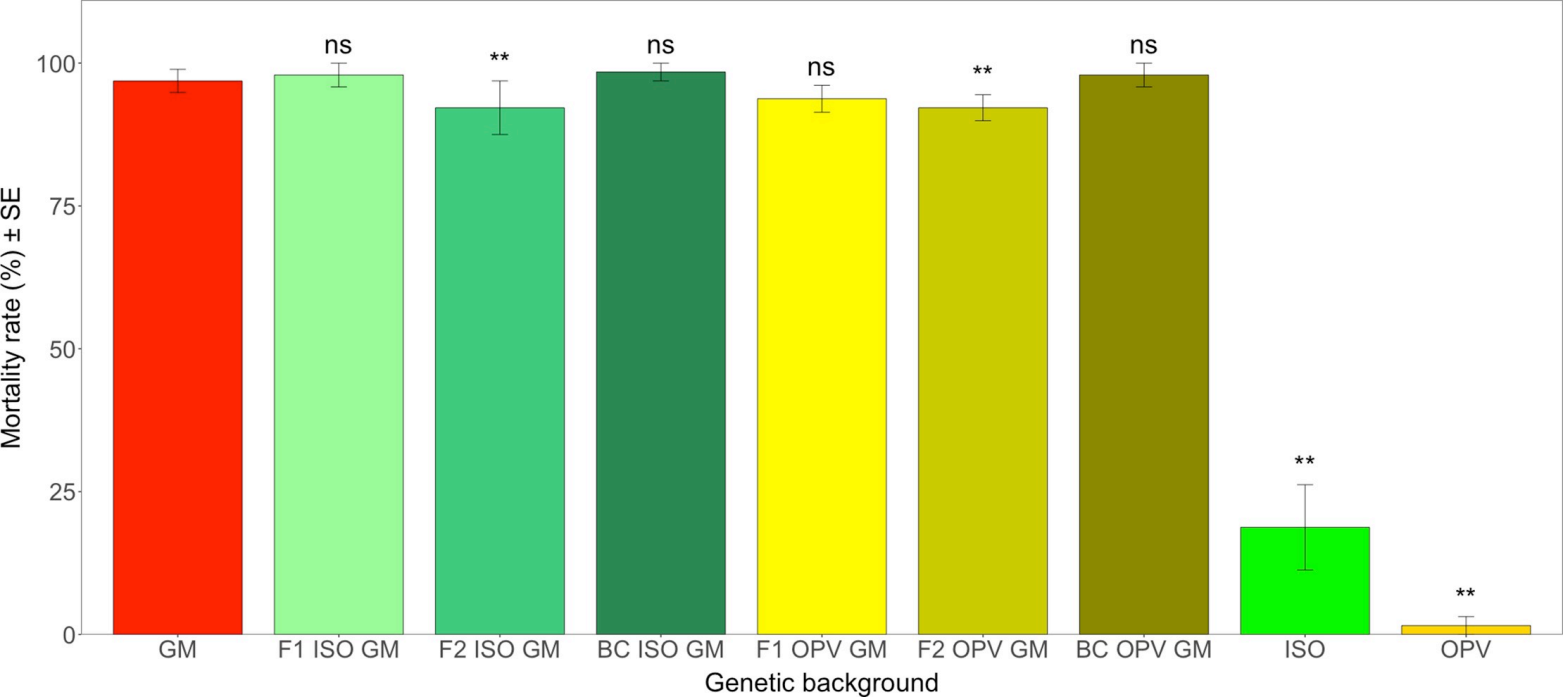
Mean mortality rate (%) of *H*. *armigera* in different genetic backgrounds from Brazil. Vertical bars show mean values, with standard error (± SE) indicated with lines. Results of multiple comparisons of means with the group control GM parental maize (Dunnett’s method). ** *P* < 0.01, ns = not significant. The p-values reported were adjusted by the single-step method.

Mean mortality rates of *H*. *armigera* larvae on all Cry1Ab maize crosses and the GM parental maize were overall high, with rates above 90% (Fig 6). The mean mortality rates of *H*. *armigera* larvae fed with materials obtained from the different genetic backgrounds from Brazil ranged between 1.56% in the non-GM OPV and 98.44% in the BC ISO GM. This led to highly significant differences of the effect of the Bt toxin crossed into the different genetic backgrounds, which is reflected in the statistical analyses (*F* = 27.913; df = 8; *P* < 0.01). There were significant differences in mortality rates between the non-GM ISO (*Z* = -6.931; *P* < 0.01) and OPV (*Z* = -6.854; *P* < 0.01) and the GM parental maize plants. However, mortality rates of *H*. *armigera* larvae did not differ between the various Cry1Ab crosses and the GM parental maize, except for F2 ISO GM (*Z* = -3.325; *P* < 0.01) and F2 OPV GM (*Z* = -5.292; *P* < 0.01) (Fig 6). For these two crosses, mortality rates of *H*. *armigera* larvae were slightly lower than for the GM parental maize. Interestingly, these two genetic backgrounds had the lowest and the highest Cry1Ab protein concentrations, respectively. However, the differences were very small and probably of minor biological relevance in a field setting.

Although considered a non-target pest to GM maize that express the Cry1Ab protein, the mortality rates of *H*. *armigera* larvae demonstrated that the produced Cry1Ab protein was bioactive inducing mortality rates above 90% across the different genetic backgrounds tested.

**Fig 7:**
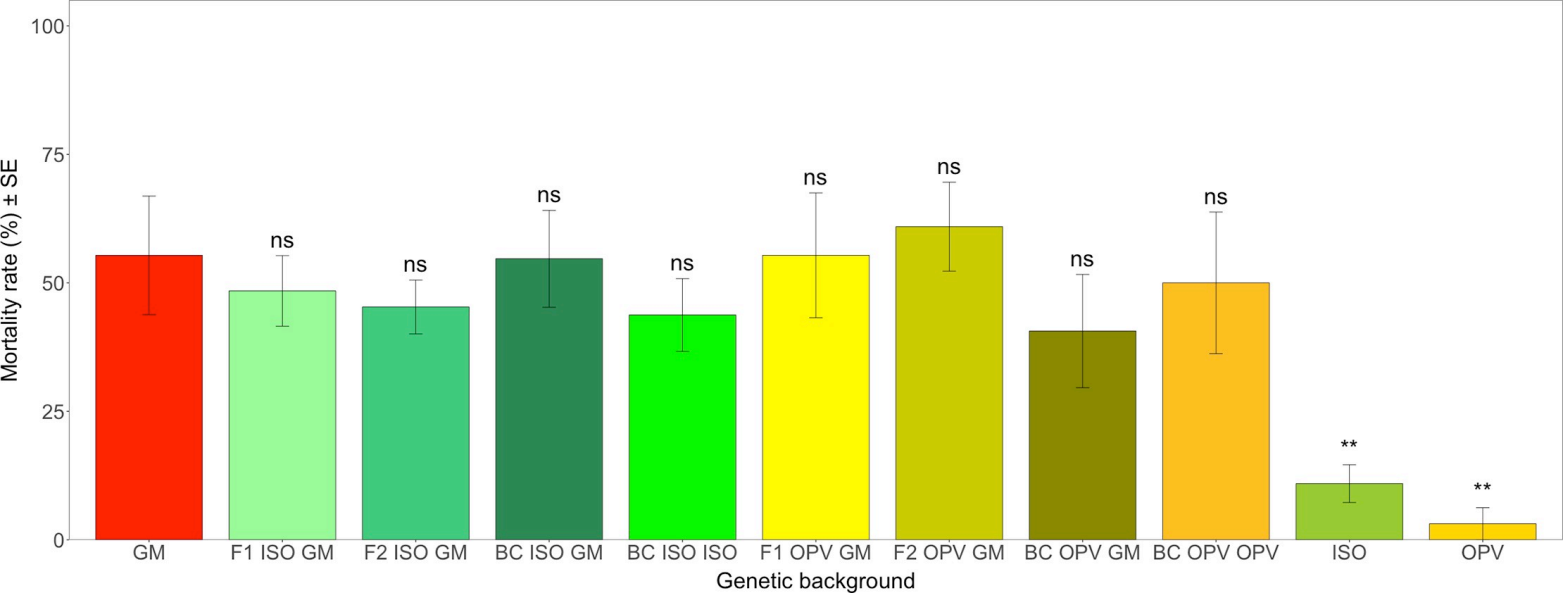
Mean mortality rate (%) of *S*. *littoralis* in different genetic backgrounds from South Africa. Vertical bars show mean values, with standard error (± SE) indicated as lines. Results of multiple comparisons of means with the group control GM parental maize (Dunnett’s method). ** *P* < 0.01, ns = not significant. The p-values reported were adjusted by the single-step method.

The mean mortality rates of *S*. *littoralis* larvae fed with the different crosses from South Africa was much lower than the toxicity observed on *H*. *armigera*, as expected from a non-target insect pest [40]. Values here ranged between 3.13% on non-GM OPV and 60.94% on F2 OPV GM leaves (Fig 7). Here again, there was a significant difference in mortality rates between different genetic backgrounds (*F* = 5.370; df = 10; *P* < 0.01), with significant differences between non-GM ISO (*Z* = -4.986; *P* < 0.01) and OPV (*Z* = -4.920; *P* < 0.01) compared to GM parental maize (Fig 7). The mortality rates of *S*. *littoralis* on all Cry1Ab crosses and the GM parental maize ranged between 40.63% and 60.94% (Fig 7). A relative insensitivity to Bt toxins in *S*. *littoralis* has been shown also for bacteria-produced Cry1 toxins by others [82, 83].

**Fig 8:**
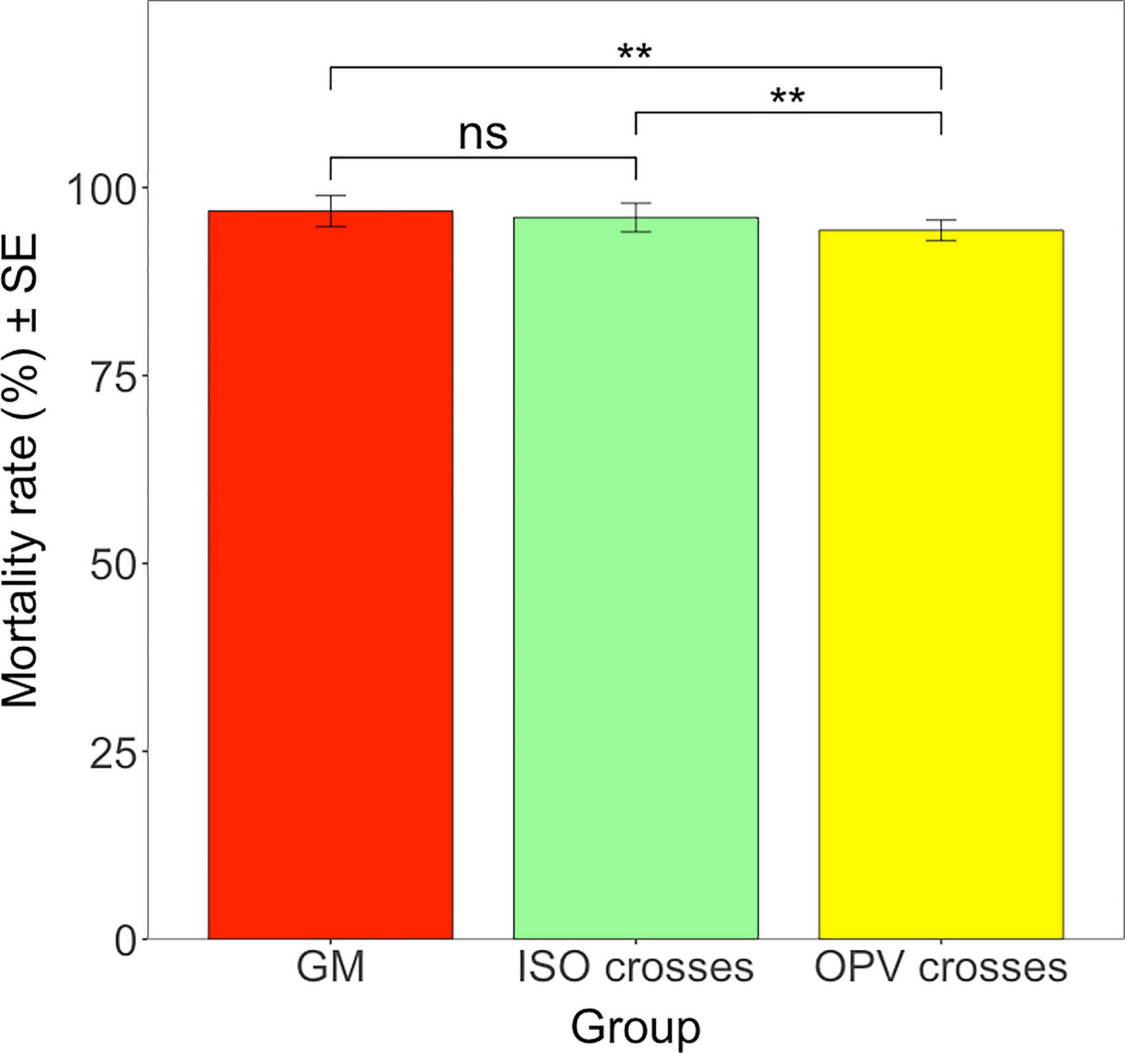
Mean mortality rate (%) of *H*. *armigera* in different groups from Brazil. Vertical bars show mean values, with standard error (± SE) indicated as lines. Results of pairwise comparisons between the different groups. ** *P* < 0.01, ns = not significant. The p-values were adjusted by the Tukey comparison method.

When data for *H*, *armigera* were pooled according to ISO and OPV crosses from Brazil, mortality rates ranged between 94.32% on OPV crosses and 96.88% on GM parental maize (Fig 8). There was a significant difference in mortality rates between the different groups (*F* = 3.534; df = 2; *P* < 0.05) but the mortality differences were small. Results showed significant differences between OPV crosses and GM parental maize (*Z* = 3.316; df = inf.; *P* < 0.01) and between ISO crosses and OPV crosses (*Z* = 3.217; df = inf.; *P* < 0.01). However, no significant difference between ISO crosses and GM parental maize was detectable (*Z* = 1.884; df = inf.; *P* = 0.143) (Fig 8).

On the ISO and OPV crosses from South Africa, mortality rates of *S*. *littoralis* ranged between 48.05% on ISO crosses and 55.36% on GM parental maize, and there was no significant difference in mortality rates between different groups (*F* = 0.360; df = 2; *P* = 0.699). Thus, mortality rates of *S*. *littoralis* on the ISO (48.05%) and OPV (51.61%) crosses from South Africa were similar to GM parental maize (55.36%).

When considering only the crosses expressing the Cry1Ab protein, the Fligner-Killeen test results revealed no significant difference in variances between the different genetic backgrounds from Brazil (*P* = 0.386) or between different groups (*P* = 0.457) in the mortality rate of *H*. *armigera*. In South African crosses, there was also no significant difference in the variances between different genetic backgrounds (*P* = 0.129). However, between different groups (GM parental, ISO crosses and OPV crosses), this test revealed a significant difference in variances (*P* < 0.05) in the mortality rate of *S*. *littoralis*.

### Correlation between mortality rate and Cry1Ab concentration

**Fig 9:**
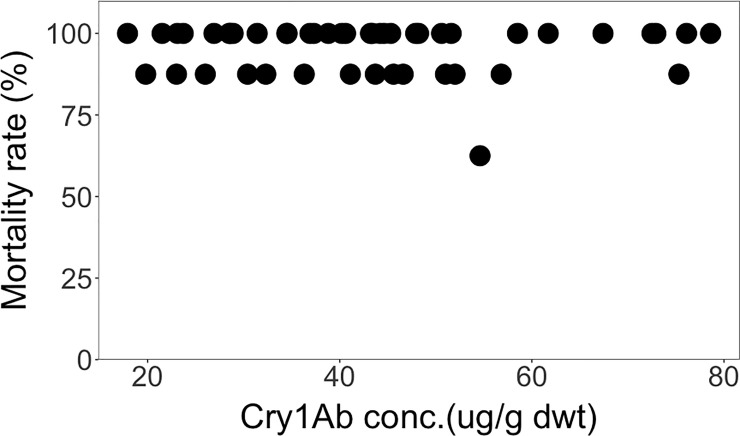
Spearman rank correlation between mortality rate and Cry1Ab concentrations across different genetic backgrounds from Brazil. (*Rs* = -0.01, *P* = 0.929).

Analysis of individual mortality rates in relation to the individually measured Cry1Ab concentrations of each leaf piece that was fed to each individual *H*. *armigera* larvae showed that all measured Cry1Ab concentrations induced fairly high mortality rates of 85 to 100%, even though the Cry1Ab concentrations varied more than 4-fold (17.9 μg/g dwt– 78.6 μg/g dwt) (Fig 9). There was no significant correlation between the Cry1Ab concentrations and the mortality rates across different genetic backgrounds (*Rs* = -0.01, *P* = 0.929) (Fig 9). It was also shown that while not all *H*. *armigera* larvae were killed by the Cry1Ab concentration expressed, their survival was not dose-dependent.

**Fig 10:**
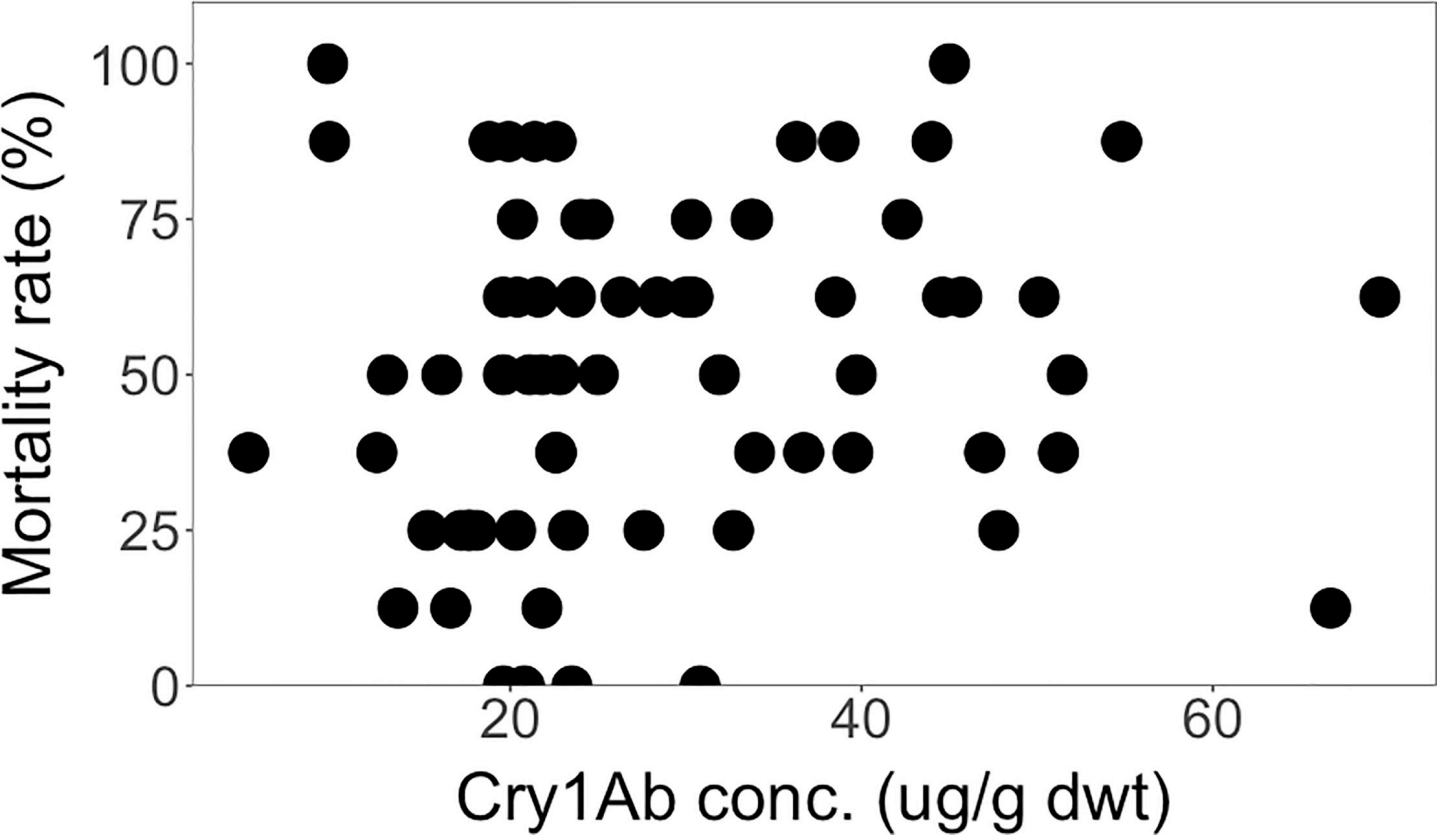
Spearman rank correlation between mortality rate and Cry1Ab concentrations across the different genetic backgrounds from South Africa. (*Rs* = 0.19, *P* = 0.12).

The measured Cry1Ab concentrations in the different genetic backgrounds from South Africa ranged between 5.1 μg/g dwt and 69.5 μg/g dwt (more than 13-fold) (Fig 10). These Cry1Ab concentrations induced mortality rates of *S*. *littoralis* larvae that varied between 0% and 100%. Again, there was no statistically significant correlation between the Cry1Ab concentrations and the mortality rates of *S*. *littoralis* larvae across the different genetic backgrounds from South Africa (*Rs* = 0.19, *P* = 0.12) (Fig 10). Interestingly, some genetic backgrounds that produced Cry1Ab protein did not produce enough to kill any of the larvae, which we attribute to a combination of low or insufficient levels of Cry1Ab and the known relative resistance of *S*. *littoralis* larvae to the protein toxin mentioned above.

Additionally, results did not show a significant correlation between Cry1Ab concentrations and mortality rates of *H*. *armigera* and *S*. *littoralis* in any genetic background or group from Brazil and South Africa, respectively (S9 and S10 Tables). Similarly, Erasmus et al. [13], using these same African genetic backgrounds but a different lepidopteran species, reported a lack of correlation between Cry1Ab concentration and *Busseola fusca* survival. Erasmus et al. [13] concluded that the effect that transgene flow to OPVs may have on resistance evolution was overestimated.

## Conclusions

To our knowledge, this is the first time that the triple relationship between gene transcription, translation into protein and bioactivity has been documented during the process of outcrossing of a transgene into various different hybrid and open-pollinating maize varieties. This contrast with the many studies focused on transgene inheritance and expression in stable, homogenous genetic backgrounds. We draw the following main conclusions from the data presented in this study:

- The *cry1Ab* transgene can successfully outcross into different maize varieties and genetic backgrounds. In our experience, most crosses, but not all, followed the expected Mendelian segregation pattern. The transgene is stably expressed in all tested genetic backgrounds, regardless of whether these maize varieties were the outcomes of a commercial hybrid breeding program or the products of farmer-selection of OPVs.
- Bt toxins associated with the insertion of the corresponding transgene through crosses were available on the plant in a bioactive form.
- Nevertheless, while transgene introgression was reliably obtained and transcription into mRNA was consistent, highly variable concentrations of Cry1Ab toxins were expressed in various crosses, similar to GM parental maize.
- No consistent correlation could be found between gene expression (measured through mRNA concentrations), protein concentrations and insect mortality.
- The specific genetic background into which the transgene was inserted played a complex role in determining the eventual expression of the transgene in field-relevant traits. Results with our South African crosses suggest an effect of background on transgene expression, possibly through zigosity or other gene-dosage effects in the transgene locus.

A surprising outcome of this study was that the transcription rates of the transgene could not be said to determine directly the concentration of the produced Cry1Ab toxin. The presence of an active transgene seems to serve only as a necessary, but not sufficient, trigger inducing the GM plant to produce the toxin, but without exercising a quantitative control over how much of this toxin will be produced. Transcription activity of the transgene, measured as the concentration of corresponding mRNA, therefore does not appear to be a reliable indicator of protein product concentration or bioactivity.

Consistently, our data showed high variability in the produced Cry1Ab concentrations (by a factor greater than four in the Brazilian crosses and greater than thirteen in those from South Africa), although we also show that even the lowest Cry1Ab concentration still induced fairly high mortality rates in *H*. *armigera* and S. *littoralis*.

The questions raised by these observations are of such consequence that further work into their causes seems crucial. Further study would reveal how general this phenomenon might be, or whether it applies only to the controlled conditions and the genetic materials we tested.

In practice, the management of pest resistance through Insect Resistance Management (IRM) strategies requires reliably high concentrations of the toxin as a key prerequisite for success, and such levels are mandatory for most countries where Bt toxin-producing crops are cultivated [5]. Our results show that the reliable delivery of expected high concentrations of toxin cannot be assumed simply from the presence of the transgene in the maize plant, casting doubt on *a priori* assumptions that IRM regimes will operate successfully. To what extent this effect is also prevalent in other crops and other methods of genetic engineering should be a priority of research, but also a cautionary consideration in all policies for adoption and management of genetically engineered crops.

Our results also apply to the intentional outcrossing of GM traits into OPVs, as in the Water Efficient Maize for Africa project (WEMA) [84] or the Insect Resistance Maize for Africa project (IRMA) in Kenya [85]. These projects have the goal to intentionally introduce an insecticidal Bt trait (like the Cry1Ab studied here) into OPVs of maize used predominantly by small-scale farmers. Our data may be reassuring that this can be achieved, as successful and stable transmission of a transgene was demonstrated with OPV crosses. However, these results apply to the tested maize varieties and may be only indicative for other maize OPVs. While suggestive, our results cannot be directly extrapolated, without further confirmation and expansion, to other GM crops or their OPVs and landrace relatives.

From the perspective of small-scale farmers who maintain and recycle their own maize seeds and intend to keep them free of transgenes, our results have very significant implications. Unintended outcrossing from GM varieties to OPVs will most likely lead to a permanent and sustained level of genetic contamination that would be close to impossible to remove, as demonstrated by our crossing experiments. The confirmation of the triple relationship between stable transgene transcription, reliable Cry1Ab toxin production and confirmed bioactivity provides evidence to expect that once unintended genetic contamination takes place in small-scale farmers’ fields, serious ecological and evolutionary consequences are to be expected. Not only is the crossing behavior of transgenes carried through pollination a source of concern, but our confirmation of bioactivity in backcrosses of OPVs suggests that farmers’ behavior will likely be influenced towards selection of GM traits, as farmers will unknowingly select crossed lines that carry the insecticidal trait, certainly in areas where lepidopteran pest damage is prevalent. The strong insecticidal trait conferred by GM crosses would conceal and underplay other traits that have previously been selected over many years. Taken together, our analyses lead us to conclude that transgene flow into OPVs and landrace varieties should be expected to have serious consequences for evolution of maize populations and seed diversity cultivated by small-scale farmers.

## Supporting information

S1 Fig Sampling scheme. (PDF)

S2 Fig Bioassay tray (32 cells/tray). (PDF)

S1 Table Mendel’s inheritance expectation, number of positive and negative GM plants observed and expected and χ^2^ calculated for different genetic backgrounds from Brazil. (PDF)

S2 Table Mendel’s inheritance expectation, number of positive and negative GM plants observed and expected and χ^2^ calculated for the different genetic backgrounds from South Africa. (PDF)

S3 Table Number total of analyzed maize seedlings in each genetic background. (PDF)

S4 Table Components of the master mix. (PDF)

S5 Table Sequences and amplification efficiencies of TaqMan primers and probes for the *cry1Ab* transgene and three reference genes in the Brazil experiment. (PDF)

S6 Table Sequences and amplification efficiencies of TaqMan primers and probes for the *cry1Ab* transgene and three reference genes in the South Africa experiment. (PDF)

S7 Table Spearman’s rank correlation and p-value between relative transgene expression and Cry1Ab concentration across different genetic backgrounds from Brazil and South Africa. (PDF)

S8 Table Spearman’s rank correlation and p-value between relative transgene expression and Cry1Ab concentration across different groups from Brazil and South Africa. (PDF)

S9 Table Spearman’s rank correlation and p-value between mortality rate and Cry1Ab concentration across different genetic backgrounds from Brazil and South Africa. (PDF)

S10 Table Spearman’s rank correlation and p-value between mortality rate and Cry1Ab concentration across different groups from Brazil and South Africa. (PDF)
