# Supplementary material for: Transgene behavior in Zea mays L. crosses across different genetic backgrounds: Segregation patterns, cry1Ab transgene expression, insecticidal protein concentration and bioactivity against insect pests
Source: PLoS One. 2020 Sep 10;15(9):e0238523. doi: 10.1371/journal.pone.0238523 (PMC7482933; doi:10.1371/journal.pone.0238523)
Supplement: S1 Fig — (PDF) [file pone.0238523.s001.pdf]

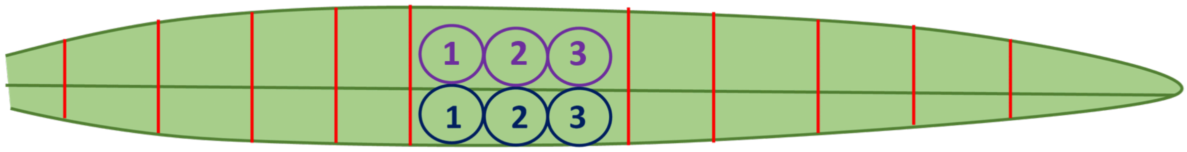

Number 1 circular samples were used for qRT-PCR analysis; number 2 and 3 for ELISA analysis; the leaf remaining was divided in eight pieces for the bioassay with *H. armigera* or *S. littoralis*. Figure credit: [37].
