# Supplementary figures and images for: Transgene behavior in Zea mays L. crosses across different genetic backgrounds: Segregation patterns, cry1Ab transgene expression, insecticidal protein concentration and bioactivity against insect pests
Source: PLoS One. 2020 Sep 10;15(9):e0238523. doi: 10.1371/journal.pone.0238523 (PMC7482933; doi:10.1371/journal.pone.0238523)

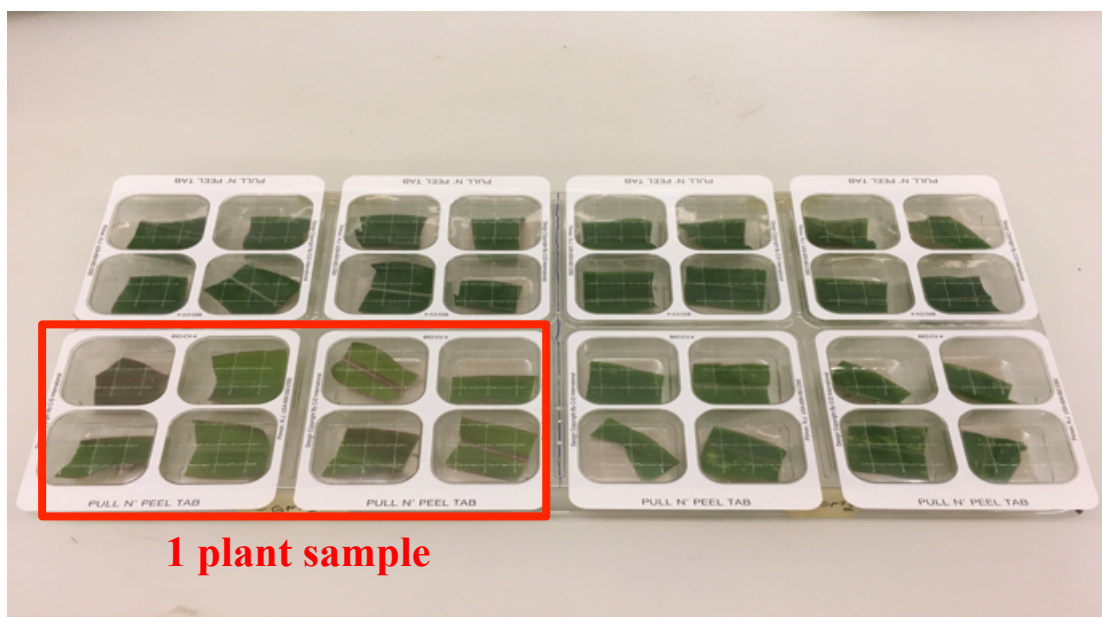

Supplement: S2 Fig — (PDF) [file pone.0238523.s002.pdf]
