# Supplementary material for: Transgene behavior in Zea mays L. crosses across different genetic backgrounds: Segregation patterns, cry1Ab transgene expression, insecticidal protein concentration and bioactivity against insect pests
Source: PLoS One. 2020 Sep 10;15(9):e0238523. doi: 10.1371/journal.pone.0238523 (PMC7482933; doi:10.1371/journal.pone.0238523)
Supplement: S1 Table — (PDF) [file pone.0238523.s003.pdf]

Genetic background	Mendel's expectation (%)		N° plants analyzed	N° obs. (+) GM plants	N° obs. (-) GM plants	N° exp. (+) GM plants	N° exp. (-) GM plants	Σ χ^2	χ^2 (0.05;1)	χ^2 (0.01;1)
	(+) GM	(-) GM								
F1 ISO GM	50	50	93	37	56	46.5	46.5	3.88	3.84	6.63
F2 ISO GM	43.75	56.25	101	31	70	44.19	56.81	7	3.84	6.63
BC ISO GM	62.5	37.5	85	46	39	53.13	31.87	2.92	3.84	6.63
F1 OPV GM	50	50	100	38	62	50	50	5.76	3.84	6.63
F2 OPV GM	43.75	56.25	116	46	70	50.75	65.25	0.79	3.84	6.63
BC OPV GM	62.5	37.5	75	26	49	46.88	28.12	24.8	3.84	6.63
BC OPV OPV	25	75	33	0	33	8.25	24.75	11	3.84	6.63
