# Supplementary material for: Transgene behavior in Zea mays L. crosses across different genetic backgrounds: Segregation patterns, cry1Ab transgene expression, insecticidal protein concentration and bioactivity against insect pests
Source: PLoS One. 2020 Sep 10;15(9):e0238523. doi: 10.1371/journal.pone.0238523 (PMC7482933; doi:10.1371/journal.pone.0238523)
Supplement: S2 Table — (PDF) [file pone.0238523.s004.pdf]

**Genetic background**	**Mendel's expectation (%)**		**N° plants analyzed**	**N° obs. (+) GM plants**	**N° obs. (-) GM plants**	**N° exp. (+) GM plants**	**N° exp. (-) GM plants**	**Σ χ^2**	**χ^2 (0.05;1)**	**χ^2 (0.01;1)**
	**(+) GM**	**(-) GM**								
F1 ISO GM	50	50	61	31	30	30.5	30.5	0.02	3.84	6.63
F2 ISO GM	75	25	70	60	10	52.5	17.5	4.28	3.84	6.63
BC ISO GM	75	25	61	46	15	45.75	15.25	0.01	3.84	6.63
BC ISO ISO	50	50	67	29	38	33.5	33.5	1.21	3.84	6.63
F1 OPV GM	50	50	84	37	47	42	42	1.19	3.84	6.63
F2 OPV GM	75	25	87	62	25	65.25	21.75	0.65	3.84	6.63
BC OPV GM	75	25	69	53	16	51.75	17.25	0.12	3.84	6.63
BC OPV OPV	50	50	78	46	32	39	39	2.51	3.84	6.63
