# Supplementary material for: Transgene behavior in Zea mays L. crosses across different genetic backgrounds: Segregation patterns, cry1Ab transgene expression, insecticidal protein concentration and bioactivity against insect pests
Source: PLoS One. 2020 Sep 10;15(9):e0238523. doi: 10.1371/journal.pone.0238523 (PMC7482933; doi:10.1371/journal.pone.0238523)
Supplement: S3 Table — (PDF) [file pone.0238523.s005.pdf]

Genetic background	N° of seedlings	
	Brazil	South Africa
GM	8	7
F1 ISO GM	6	7
F2 ISO GM	8	8
BC ISO GM	8	8
BC ISO ISO	-	8
F1 OPV GM	8	8
F2 OPV GM	8	8
BC OPV GM	6	8
BC OPV OPV	-	8
ISO	8	8
OPV	8	8
**Total**	**68**	**86**
