# Supplementary material for: Transgene behavior in Zea mays L. crosses across different genetic backgrounds: Segregation patterns, cry1Ab transgene expression, insecticidal protein concentration and bioactivity against insect pests
Source: PLoS One. 2020 Sep 10;15(9):e0238523. doi: 10.1371/journal.pone.0238523 (PMC7482933; doi:10.1371/journal.pone.0238523)
Supplement: S4 Table — (PDF) [file pone.0238523.s006.pdf]

Reagent	Volume (µl)
Forward primer (3µM)	1.1
Reverse primer (3µM)	1.1
Probe (5µM)	0.5
TaqMan® gene expression Mix	5
MiliQ H_2O	2.3
**Total**	**10**
