# Supplementary material for: Transgene behavior in Zea mays L. crosses across different genetic backgrounds: Segregation patterns, cry1Ab transgene expression, insecticidal protein concentration and bioactivity against insect pests
Source: PLoS One. 2020 Sep 10;15(9):e0238523. doi: 10.1371/journal.pone.0238523 (PMC7482933; doi:10.1371/journal.pone.0238523)
Supplement: S5 Table — (PDF) [file pone.0238523.s007.pdf]

Name	Sequence	PCR efficiency	Reference
cry1Ab		1.811	A. Coll
for	5'-GCTCATCAACCAGAGGATCGA-3'		
rev	5'-AGCGTAGATTTGGTAGAGGTTGCT-3'		
probe	5' FAM-TCGCCAGGAACCAGGCCATCAG-TAMRA3'		
lug		1.790	[38]
for	5'-GAAAACACACGAGTGGAATTGATT-3'		
rev	5'-CGGTCAGAATATGGTCATTCAGTT-3'		
probe	5' FAM-CGCTGCGATTCAATTCATGCACAAA-TAMRA3'		
mep		1.798	[38]
for	5'-CCCACTGGGTTGCAATCCT-3'		
rev	5'-GGTGAGAGCGGAAAGCTTGT-3'		
probe	5' FAM-ACCCAGACAGATGGCCACCCAACAT-TAMRA3'		
ubcp		1.749	[38]
for	5'-ATCCTCTTGTCCCTGAGATTGC-3'		
rev	5'-AGCGTGCGGTGGACTCATA-3'		
probe	5' FAM-CACATGTACAAGACCGACAGGGCCAA-TAMRA3'		
