# Supplementary material for: Transgene behavior in Zea mays L. crosses across different genetic backgrounds: Segregation patterns, cry1Ab transgene expression, insecticidal protein concentration and bioactivity against insect pests
Source: PLoS One. 2020 Sep 10;15(9):e0238523. doi: 10.1371/journal.pone.0238523 (PMC7482933; doi:10.1371/journal.pone.0238523)
Supplement: S7 Table — (PDF) [file pone.0238523.s009.pdf]

**Genetic background**	***P***		**Spearman's correlation (*Rs*)**	
	**Brazil**	**South Africa**	**Brazil**	**South Africa**
GM	0.50	0.33	-0.29	0.80
F1 ISO GM	0.75	0.08	0.40	-1.00
F2 ISO GM	0.66	0.79	0.26	0.12
BC ISO GM	0.17	**0.007**	0.61	**0.88**
BC ISO ISO	-	0.43	-	0.33
F1 OPV GM	0.45	0.10	-0.50	0.77
F2 OPV GM	0.92	**0.046**	0.09	**0.74**
BC OPV GM	0.75	0.66	0.40	-0.19
BC OPV OPV	-	0.24	-	-0.47
