# Supplementary material for: Transgene behavior in Zea mays L. crosses across different genetic backgrounds: Segregation patterns, cry1Ab transgene expression, insecticidal protein concentration and bioactivity against insect pests
Source: PLoS One. 2020 Sep 10;15(9):e0238523. doi: 10.1371/journal.pone.0238523 (PMC7482933; doi:10.1371/journal.pone.0238523)
Supplement: S8 Table — (PDF) [file pone.0238523.s010.pdf]

Group	*P*		Spearman's correlation (*Rs*)	
	**Brazil**	**South Africa**	**Brazil**	**South Africa**
GM	0.50	0.33	-0.29	0.80
ISO crosses	0.32	0.19	0.26	0.25
OPV crosses	0.44	0.87	-0.18	-0.03
