# Supplementary material for: Transgene behavior in Zea mays L. crosses across different genetic backgrounds: Segregation patterns, cry1Ab transgene expression, insecticidal protein concentration and bioactivity against insect pests
Source: PLoS One. 2020 Sep 10;15(9):e0238523. doi: 10.1371/journal.pone.0238523 (PMC7482933; doi:10.1371/journal.pone.0238523)
Supplement: S9 Table — (PDF) [file pone.0238523.s011.pdf]

**Genetic background**	***P***		**Spearman's correlation (*Rs*)**	
	**Brazil**	**South Africa**	**Brazil**	**South Africa**
GM	0.77	0.36	0.13	0.41
F1 ISO GM	0.16	0.63	0.65	0.22
F2 ISO GM	1.00	0.18	0.00	0.53
BC ISO GM	0.55	0.80	0.25	-0.11
BC ISO ISO	-	0.93	-	-0.04
F1 OPV GM	0.07	1.00	-0.72	0.00
F2 OPV GM	0.69	0.66	-0.20	0.18
BC OPV GM	0.80	0.33	0.13	0.40
BC OPV OPV	-	0.93	-	0.04
