# Supplementary material for: Transgene behavior in Zea mays L. crosses across different genetic backgrounds: Segregation patterns, cry1Ab transgene expression, insecticidal protein concentration and bioactivity against insect pests
Source: PLoS One. 2020 Sep 10;15(9):e0238523. doi: 10.1371/journal.pone.0238523 (PMC7482933; doi:10.1371/journal.pone.0238523)
Supplement: S10 Table — (PDF) [file pone.0238523.s012.pdf]

**Group**	***P***		**Spearman's correlation (*Rs*)**	
	**Brazil**	**South Africa**	**Brazil**	**South Africa**
GM	0.77	0.36	0.13	0.41
ISO crosses	0.70	0.28	0.09	0.21
OPV crosses	0.16	0.54	-0.35	0.12
